# Prevalence and distribution of psychological diagnoses and related frequency of consultations in Norwegian urban general practice

**DOI:** 10.1080/02813432.2020.1783477

**Published:** 2020-06-27

**Authors:** Mina Piiksi Dahli, Mette Brekke, Torleif Ruud, Ole Rikard Haavet

**Affiliations:** a Faculty of Medicine, Institute of Health and Society, University of Oslo, Oslo, Norway;; b General Practice Research Unit, Faculty of Medicine, Institute of Health and Society, University of Oslo, Oslo, Norway;; c Division of Mental Health Services, Akershus University Hospital, Lørenskog, Norway;; d Faculty of Medicine, Institute of Clinical Medicine, University of Oslo, Oslo, Norway

**Keywords:** General practice, general urban practice, mental health, psychological diagnoses, ICPC-2, frequency of consultations

## Abstract

**Objective:** To investigate the prevalence and distribution of psychological diagnoses made by general practitioners (GPs) in urban general practice and the related frequency of consultations during 12 consecutive months in Norwegian general practice.

**Design:** A cross-sectional study with data extracted from 16,845 electronic patient records in 35 urban GP practices

**Setting:** Six GP group practices in Groruddalen, Norway.

**Subjects:** All patients aged 16–65 with a registered contact with a GP during 12 months in 2015.

**Main outcome measures:** Frequency and distribution of psychological diagnoses made by GPs, and the number of patients’ consultations.

**Results:** GPs made a psychological diagnosis in 18.8% of the patients. The main diagnostic categories were depression symptoms or disorder, acute stress reaction, anxiety symptoms or disorder and sleep disorder, accounting for 67.1% of all psychological diagnoses given. The mean number of consultations for all patients was 4.09 (95% CI: 4.03, 4.14). The mean number of consultations for patients with a psychological diagnosis was 6.40 (95% CI: 6.22, 6.58) compared to 3.55 (95% CI 3.50, 3.51) (*p*<0.01) for patients without such a diagnosis. Seven percent of the diagnostic variation was due to differences among GPs.

**Conclusions:** Psychological diagnoses are frequent in urban general practice, but they are covered using rather few diagnostic categories. Patients with psychological diagnoses had a significantly higher mean number of GP consultations regardless of age and sex.

**Implications:** The knowledge of the burden of psychological health problems in general practice must be strengthened to define evidence-based approaches for detecting, diagnosing and treating mental disorders in the general practice population.

**Key Points**
- Eighteen percent of patients aged 16–65 in our study of patients in urban general practice received one or more psychological diagnoses in 12 months.
- Depression was the most common diagnosis; followed by acute stress reaction, anxiety and sleep disturbance.
- Patients with psychological diagnoses had a significantly higher mean number of consultations compared to patients without such diagnoses regardless of age and sex.

## Introduction

Mental disorders are among the most common chronic health disorders worldwide [1]. These patients have lower life quality, lower life expectancy and higher disability-adjusted life years compared to the general population [2–5]. This poses a burden on health- and welfare systems, especially on the primary health care services where most of these disorders are treated [6,7]. Studies show a varying prevalence of mental disorders in general practice both in urban, suburban and rural settings. A large Danish study found that general practitioners (GPs) classified 11% of their patients with psychological problems [8]. A Spanish study found that the 12-month prevalence of any mental disorder in general practice was 23% [2]. A questionnaire survey with over 2000 participants from general practice in Belgium found a mental disorder in 42.5% of all patients, although only 5.4% of the patients consulted their doctor for mental health problems [9].

Patients with mental disorders seem to contact their GP more often than patients without such disorders [10,11]. However, studies on the impact of mental health problems on GPs’ workload are few and show varying results. A Norwegian study based upon direct observation in urban general practice found that about every fourth of primary care consultations deals with a psychological problem [12]. A Danish study found that 2% of the working-age population contacted their GP during a six-month period for psychological stress [13].

There have been concerns that current research and treatment models for mental disorders do not adequately address the complex challenges of mental illness as it is presented in general practice [14] and there have been suggestions to move into more collaborative-type care models [15].

### Aim

In this study, we wanted to investigate the scope of GP’s work with mental disorders by studying the prevalence and distribution of GP assessed psychological diagnoses and the related frequency of consultations in a Norwegian urban setting. We wanted to assess if patients with psychological diagnoses consult more frequently compared to patients without such diagnoses, and how these matters vary with patients’ age and sex, and between individual GPs.

## Materials and methods

### Design

A cross-sectional study during 12 consecutive months.

### Setting

The recruitment was part of a larger cluster-randomized controlled study; Shared Care and Usual Health Care for Mental and Comorbid Health Problems [16]. For this study, we recruited two GP office centers each from three boroughs Grorud, Stovner and Alna in Groruddalen in Oslo, Norway. The recruitment followed the principles of availability sampling and the order of invitation was by equality in size between the boroughs (offices with 4–6 GPs were prioritized before offices with one, two or more than six GPs). The centers were contacted by telephone, followed by a visit including several members of the research group to introduce the project. The GPs would then decide on participation before signing a detailed contract. Nine GP office centers were contacted before six agreed to participate.

### Data

Norwegian GPs record all medical contacts electronically, in order to obtain reimbursement in a government-aided tariff system. The International Classification of Primary Care 2 (ICPC-2) is used for diagnosis coding [17]. ICPC-2 divides into chapters that cover medicine at large, where chapter ‘P – psychological’ – consists of 26 codes for mental health complaints and 17 codes for psychiatric diagnoses. In this article, the term ‘psychological diagnoses’ covers all of these diagnostic codes. Outcome measures were psychological diagnoses made by the participating GPs during the 12 months period, as well as the number of consultations for their patients with or without such diagnoses. The electronic medical records from 17,973 patients and 111,870 contacts were extracted. Of these, 16,845 patients had one or more consultations with their GP, either in the form of office- or home visit. These accounted for 68,814 contacts during the 12 months, and these form the sample used in this study. The remaining excluded contacts were either a phone call, letter, prescription or interdisciplinary meetings. Contacts without a registered ICPC-2 diagnostic code were excluded.

### Data collection

We extracted data from all contacts by all patients between 16 and 65 years of age seen by any of the participating GPs during 12 consecutive months in 2014 and 2015. There were no exclusion criteria. Variables extracted were age, sex, date of contact, type of contact, ICPC-2 diagnoses and reimbursement codes. A computer program was developed by the firm Mediata AS for this project to extract data from the different electronic medical records in the GP office centers.

### Data analyses

Descriptive statistics in the form of frequencies (*n*) and percentages (%) were used to explore the distribution of psychological diagnoses. The number of consultations was described by means and standard deviations (mean ± SD). Differences in the mean number of consultations between participants with and without psychological diagnoses were analyzed using the independent *t*-test. Binary responses relating to whether a patient was given a psychological diagnosis or not by their GP were analyzed using a binary logistic regression model. In particular, we used the multilevel binary logistic regression to account for data clustering at the GP level. We obtained estimates of odds ratios (ORs) and their 95% confidence intervals from the model that was adjusted for patients’ age and sex. In addition, we also obtained an estimate of the intra-cluster correlation (ICC) from the adjusted model. Here, the ICC described the variation in giving a psychological diagnosis that was attributable to differences between the GPs. All analyses were performed using Stata SE 15 (StataCorp, College Station, TX) and IBM SPSS Statistics 25 (Armonk, NY) and the significance level was set at *p*=.05.

### Ethics

The project was approved by the Regional Committee on Medical and Health Research Ethics Health Region South East (reg. no. 2014/435), by the National Committee on Medical and Health Research Ethics (reg. no. 2014/160) and by the Data Protection Officer at Akershus University Hospital, Oslo (reg. no. 13/138).

## Results

### Patient characteristics

There were 9613 (57%) women and 7237 (43%) men in the sample. Mean age for the whole sample was 40.13 (95% CI: 39.93, 40.34) years, 39.90 (95% CI: 39.63, 40.16) years for women and 40.45 (95% CI: 40.13, 40.77) years for men. Women accounted for 42,992 (62.5%) and men 25,822 (37.5%) of the consultations.

### GP characteristics

There were 35 GPs included in this study. Eighteen were women and 17 were men. The mean age for the whole group was 50.43 (95% CI: 46.84, 54.01). Mean age for women was 49.93 (95% CI: 44.94, 53.73) years and for men 51.59 (95% CI: 45.38, 57.79) years. There were 28 (80.0%) specialists in family medicine and seven (20.0%) non-specialists; 15 of the 18 women (87.5%) and 13 of the 17 men (76.8%) were specialists.

### Psychological diagnoses

Of the 68,814 consultations, 9582 resulted in psychological diagnosis, accounting for 13.9% of the total number of consultations. The women had 5947 (62.1%) and men 3635 (37.9%) of the consultations resulting in psychological diagnosis, and this accounted for 16.1% and 16.4% of the total number of consultations for women and men, respectively.

18.8% of the patients received one or more psychological diagnoses (Table 1). There were 588 patients with two psychological diagnoses, 162 with three, 27 patients with four, four patient with five and one patient with six different psychological diagnoses given during the 12 months.

**Table 1.** Distribution of 4176 ICPC-II psychological diagnoses (mental health complaints and psychological diagnoses) in 3162 patients aged 16–65 years during 12 months.

ICPC-2 code	Diagnosis	Women, *N* (%)	Men, *N* (%)	Sum, *N* (%)
P01	Feeling anxious/nervous/tense	138 (3.30)	70 (1.68)	208 (4.98)
P02	Acute stress reaction	463 (11.09)	158 (3.78)	621 (14.87)
P03	Feeling depressed	164 (3.93)	86 (2.06)	250 (5.99)
P04	Feeling/behaving irritable/angry	2 (0.05)	2 (0.05)	4 (0.10)
P05	Senility feeling/behaving old	1 (0.02)	4 (0.10)	5 (0.12)
P06	Sleep disturbance	262 (6.27)	187 (4.48)	449 (10.75)
P07	Sexual desire reduced	0 (0.00)	4 (0.10)	4 (0.10)
P08	Sexual fulfillment reduced	1 (0.02)	50 (1.20)	51 (1.22)
P09	Sexual preference concern	2 (0.05)	4 (0.10)	6 (0.14)
P10	Stammering/stuttering/tic	2 (0.05)	3 (0.07)	5 (0.12)
P11	Eating problem in child	2 (0.05)	0 (0.00)	2 (0.05)
P15	Chronic alcohol abuse	17 (0.41)	51 (1.22)	68 (1.63)
P16	Acute alcohol abuse	1 (0.02)	2 (0.05)	3 (0.07)
P17	Tobacco abuse	32 (0.77)	51 (1.22)	83 (1.99)
P18	Medication abuse	16 (0.38)	11 (0.26)	27 (0.65)
P19	Drug abuse	11 (0.26)	46 (1.10)	57 (1.36)
P20	Memory disturbance	35 (0.84)	29 (0.69)	64 (1.53)
P22	Child behavior symptom/complaint	4 (0.10)	5 (0.12)	9 (0.22)
P23	Adolescent behavior symptom/complaint	1 (0.02)	3 (0.07)	4 (0.10)
P24	Specific learning problem	5 (0.12)	9 (0.22)	14 (0.34)
P27	Fear of mental health disorder	2 (0.05)	4 (0.10)	6 (0.14)
P28	Limited function/disability	4 (0.10)	2 (0.05)	6 (0.14)
P29	Psychological symptom/complaint other	208 (4.98)	125 (2.99)	333 (7.97)
P70	Dementia	6 (0.14)	4 (0.10)	10 (0.24)
P71	Organic psychosis other	1 (0.02)	1 (0.02)	2 (0.05)
P72	Schizophrenia	28 (0.67)	57 (1.36)	85 (2.04)
P73	Affective psychosis	39 (0.93)	21 (0.50)	60 (1.44)
P74	Anxiety disorder/anxiety state	228 (5.46)	126 (3.02)	354 (8.48)
P75	Somatization disorder	16 (0.38)	9 (0.22)	25 (0.60)
P76	Depressive disorder	612 (14.66)	304 (7.28)	916 (21.93)
P77	Suicide/suicide attempt	2 (0.05)	1 (0.02)	3 (0.07)
P78	Neuraesthenia/surmenage	9 (0.22)	1 (0.02)	10 (0.24)
P79	Phobia/compulsive disorder	40 (0.96)	25 (0.60)	65 (1.56)
P80	Personality disorder	14 (0.34)	23 (0.55)	37 (0.89)
P81	Hyperkinetic disorder	29 (0.69)	34 (0.81)	63 (1.51)
P82	Post-traumatic stress disorder	59 (1.41)	59 (1.41)	118 (2.83)
P85	Mental retardation	28 (0.67)	26 (0.62)	54 (1.29)
P86	Anorexia nervosa/bulimia	6 (0.14)	1 (0.02)	7 (0.17)
P98	Psychosis NOS/other	10 (0.24)	13 (0.31)	23 (0.55)
P99	Psychological disorders, other	29 (0.69)	36 (0.86)	65 (1.56)

The ICPC-2 diagnostic codes divide between symptom categories (P01–P29) and disease categories (P70–P99). Symptom diagnoses alone were given to 48.4% of the patients, disease diagnoses alone were given to 37.0% of the patients, and both symptom and disease diagnoses were given to 14.5% of the patients.

Depression was the most common diagnosis. Depressive disorder (P76) and depressive symptoms (P03) together accounted for 27.9% of all diagnoses given. Acute stress reaction (P02) was the second-largest diagnosis with 14.9% of diagnoses given. Anxiety disorder (P74) and anxiety symptoms (P01) together accounted for 13.5%, and sleep disturbance (P06) accounted for 10.8% of the total psychological diagnoses given.

### Number of consultations

**Figure 1.**
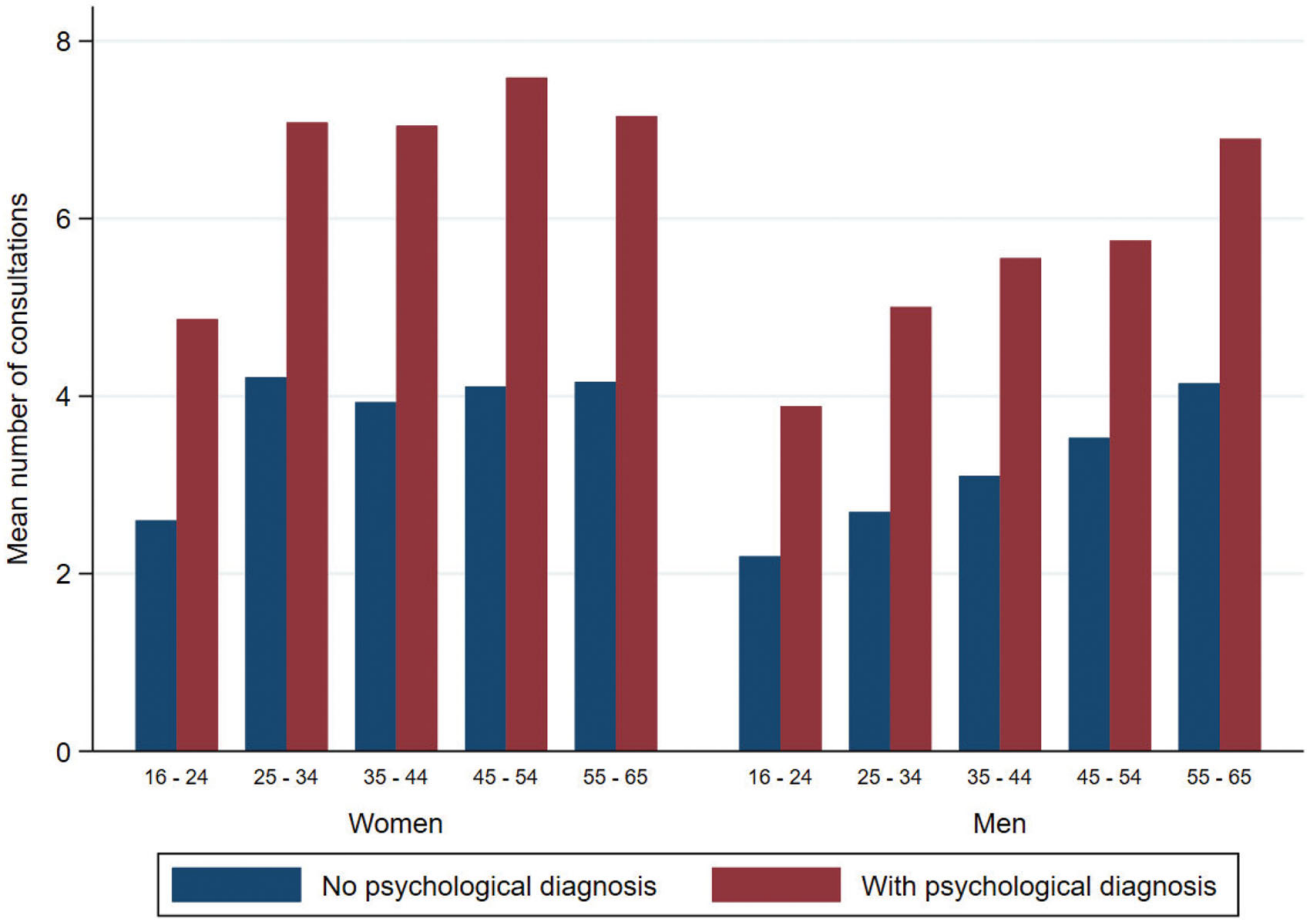
The mean number of consultations according to sex and age group for 16,845 patients aged 16–65 years with or without a psychological diagnosis during 12 months.

The number of consultations during the 12 months ranged from 1 (4373 patients) to 86 (one patient). The mean number of consultations per patient was 4.09 (95% CI: 4.03, 4.14). The mean number of consultations for women was 4.47 (95% CI: 4.39, 4.55) and for men 3.57 (95% CI: 3.49, 3.65) (Figure 1). The mean number of consultations for patients with a psychological diagnosis was 6.40 (95% CI: 6.22, 6.58) and for patients without such a diagnosis 3.55 (95% CI 3.50, 3.51) (Table 2).

**Table 2.** Mean number of consultations according to sex and age group, with or without a psychological diagnosis in 16,845 patients aged 16–65 years during 12 months.

	Overall		Mental health diagnosis		No mental health diagnosis		
	*N*	Mean ± SD	*N*	Mean ± SD	*N*	Mean ± SD	*p* Value*
Sex							
Female	9613	4.47 ± 4.06	1915	6.95 ± 5.38	7698	3.86 ± 3.40	<0.01
Male	7232	3.57 ± 3.61	1247	5.55 ± 5.38	5985	3.16 ± 3.21	<0.01
Age groups							
16–24	2680	2.70 ± 2.49	373	4.47 ± 3.74	2307	2.42 ± 2.08	<0.01
25–34	3648	4.09 ± 3.91	648	6.28 ± 5.50	3000	3.62 ± 3.29	<0.01
35–44	3797	4.17 ± 3.78	767	6.47 ± 4.74	3030	3.59 ± 3.25	<0.01
45–54	3576	4.47 ± 4.24	758	6.83 ± 5.11	2818	3.83 ± 3.73	<0.01
55–65	3144	4.72 ± 4.30	616	7.06 ± 5.67	2528	4.15 ± 3.67	<0.01

* Unadjusted *p* values (mean difference in visits for patients with vs. without psychological diagnoses).

There were 2388 (14.2%) of patients with eight or more consultations during the 12 months. The probability of having a psychological diagnosis in this group was 41.2%, compared to 15.1% (*p*<0.01) for patients with less than eight consultations.

The top 10% of attenders (1 902) accounted for 23,909 (34.7%) of the consultations, with 12.57 (95% CI: 12.36, 12.78) mean number of consultations, compared to the bottom 90% with 44,905 (65.3%) of the consultations and 3.01 (95% CI: 2.97, 3.04) mean number of consultations. The probability of having a psychological diagnosis among the top 10% of attenders was 43.3%, compared to 15.7% for the bottom 90% of attenders (*p*<0.01).

### Variation in probability for psychological diagnoses

The patients visited with their own assigned GP in 70.2% of the consultations. The remaining 31.5% were with other doctors at their GP office center, such as other GPs, substitutes or interns. Including only consultations between patients and their assigned GP, these accounted for 14,111 patients, 8160 (57.8%) women and 5951 (42.2%) men. The probability for a psychological diagnosis was 18.6% among this subgroup, 19.7% for women and 18.6% for men.).

Table 3 shows the odds of receiving a psychological diagnosis from a GP by patient sex and different age groups. Overall, the odds of receiving a psychological diagnosis were significantly higher by increasing age group compared to the 16–24 age group, with 32% in the 25–34, 56% in the 35–44, 67% in the 45–54 and 51% in the 55–65 age groups, respectively. Men were 17% less likely to receive a psychological diagnosis than women were.

**Table 3.** Estimates of ORs and their 95% CIs obtained from the multilevel binary logistic regression showing the association between patient factors and having a psychological diagnosis given.

Covariates	Unadjusted		Adjusted	
	OR (95% CI)	*p* Value	OR (95% CI)	*p* Value
Age groups (ref: 16–24)				
25–34	1.34 (1.16, 1.53)	<0.01	1.32 (1.15, 1.52)	<0.01
35–44	1.57 (1.37, 1.79)	<0.01	1.56 (1.36, 1.78)	<0.01
45–54	1.66 (1.45, 1.91)	<0.01	1.67 (1.46, 1.91)	<0.01
55–65	1.51 (1.31, 1.73)	<0.01	1.51 (1.31, 1.73)	<0.01
Sex (ref: women)				
Men	0.84 (0.77, 0.91)	<0.01	0.83 (0.77, 0.90)	<0.01

**Figure 2.**
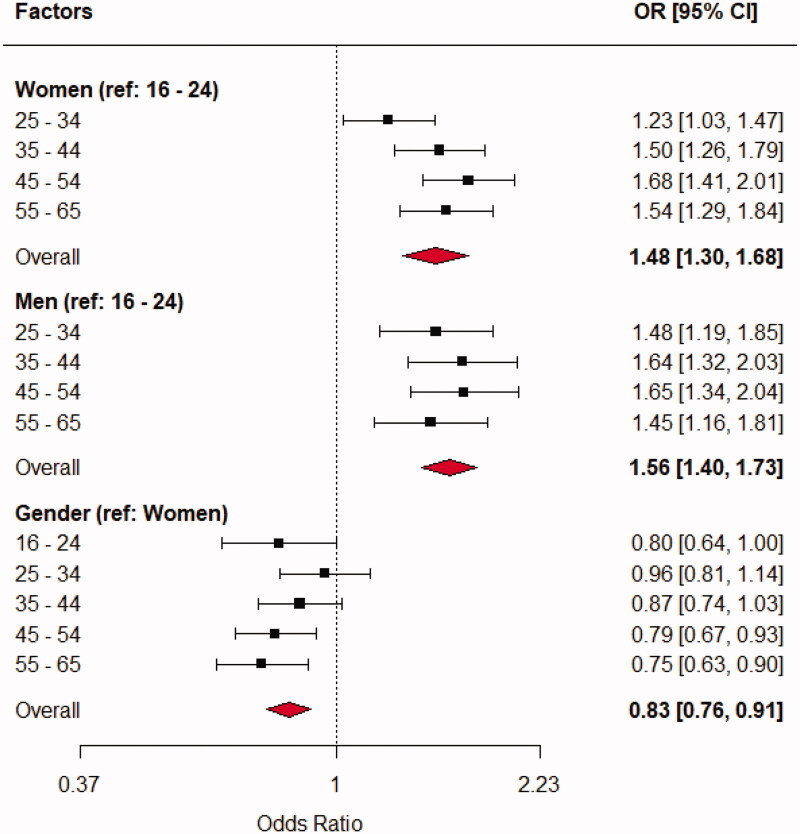
Odds of receiving a psychological diagnosis as a function of sex and age groups for 16,845 patients aged 16–65 years with or without a psychological diagnosis during 12 months.

The results of a two-way interaction between sex and age groups are presented in Figure 2. The results showed that both women and men in older age groups were more likely to receive a psychological diagnosis than patients in the age group 16–24. Overall, a woman was 48% and men 56% more likely to receive a psychological diagnosis compared to the respective 16–24 age groups.

We obtained an ICC estimate of 0.074 from the adjusted multilevel logistic regression model. This means that 7.4% of the variability of the psychological diagnoses can be attributed to differences between the GPs.

## Discussion

### Summary of findings

Eighteen percent of patients in our sample received one or more psychological diagnoses during the 12 months. Fourteen percent of the consultations resulted in psychological diagnosis. Depression symptoms or disorder (P03, P76) were the biggest diagnostic categories, followed by acute stress reaction (P02), anxiety symptoms or disorder (P01, P74) and sleep disturbance (P06). These six diagnostic categories covered 67.1% of all the diagnoses given.

Patients who received a psychological diagnosis had a significantly higher number of consultations than patients without such a diagnosis (*p*<0.001). The probability of a psychological diagnosis increased with the number of consultations. Little over 7% of the variability of the psychological diagnoses were attributed to differences between individual GPs. The odds for a psychological diagnosis was higher for the women than the men in the sample, and the odds for psychological diagnosis for the whole sample increased with increasing age.

### Discussion of results and existing literature

A report from Statistics Norway in 2017 showed psychological diagnoses to be the most frequent, with 12% of all diagnoses given to patients in Norwegian general practice [18]. This is similar but somewhat lower than in this study. The numbers from Statistics Norway are a national registry, includes all age groups, also children, and they only include the first registered diagnosis from each consultation with the GP. We know that GPs often register more than one diagnosis during each consultation, in our material, we have included all the diagnoses given during each consultation, and the study is performed in an urban setting.

The majority of psychological diagnoses in the material were captured using just a few diagnostic categories. This corresponds well with a Danish study that found that two problems (depression and acute stress reaction) accounted for 51% of all psychological classifications made in Danish general practice [8]. Some argue that the established classification systems for mental health issues are not effective and do not improve outcomes in clinical practice [19]. This may be especially true in general practice [20] where mental health issues are common but not always labeled, due to mild or passing symptoms that may not develop into more severe mental illness [21,22].

Patients who received a psychological diagnosis had a mean of 6.4 consultations during the year, compared to 3.6 for patients without a psychological diagnosis. This is higher than the total Norwegian population with an average of 2.5 yearly consultations in the 16–66 year age group in 2017 [18]. This corresponds well with other literature showing that patients with mental health issues see their doctor more frequently than patients without these issues [10,11].

The number of female patients in the material is higher than male patients, and the women see their doctor more often than the men, which results in higher absolute numbers of psychological diagnoses, consultations and we also see a higher probability for a psychological diagnosis when visiting a GP compared to the men in this material.

### Strengths and limitations

This is to our knowledge the first comprehensive study of all psychological diagnoses by GPs through 12 months in Norwegian urban general practice. We found robust trends in our results, with statistically significant variations between groups. As Norway is a country where 99% of the population are listed with a GP and less than 2% of the population change their GP during a year [23], as well as the fact that diagnoses are the basis for reimbursement for the GPs from the government, we can trust the data as reliable.

A limitation of our study is that the diagnoses itself will not give a comprehensive description of the mental health issues among patients. Sometimes, GPs will not recognize a patient’s mental health problem [24,25]. We know that patients bring up several issues during their consultations and that GPs do not always put a psychological diagnosis to all the problems addressed during a consultation [17,20]. The GP may choose not to use psychological diagnoses, due to mild symptoms and expected swift recovery, or due to stigma towards these issues [21,26]. The chance of detecting mental illness is found to increase with the number of GP consultations the patient has [27,28] and with the degree of continuity in the doctor–patient relationship [29]. This limits the generalizability of the information. There is a risk that psychological distress has been addressed in addition to other issues without a diagnosis, leading to under-reporting.

Another limitation is the geographical area of recruitment from just three boroughs in Oslo, with its distinctive urban features, in this case including a high number of immigrants and low socio-economical features. This means the population may not be representative of the population of Norway as a whole. This study also addresses the adolescent and adult population of patients, not including children under the age of 16 or the seniors above 65. If the study included more rural areas of Norway the results could be more representative for the total population.

We lack contributing factors such as socioeconomic features, ethnicity, lifestyle, alcohol and other drug use (unless there is a diagnosis of these issues) and other variables that could have further described the patients with psychological diagnoses in the material.

### Conclusions and implications

This study addresses the importance of studying psychological health problems in general practice, where the population is different from specialized health care. Patients often present complex issues or also distress in the early stages, and this will look different from traditional psychiatric illness in specialized mental health care. The knowledge of the burden of mental health problems in general practice must be strengthened to define evidence-based approaches for detecting, diagnosing and treating mental disorders in the general practice population.
